# *Zic4*-Lineage Cells Increase Their Contribution to Visual Thalamic Nuclei during Murine Embryogenesis If They Are Homozygous or Heterozygous for Loss of *Pax6* Function

**DOI:** 10.1523/ENEURO.0367-18.2018

**Published:** 2018-10-23

**Authors:** Ziwen Li, Thomas Pratt, David J. Price

**Affiliations:** 1 Simons Initiative for the Developing Brain, Biomedical Sciences, The University of Edinburgh, Edinburgh EH8 9XD, United Kingdom

**Keywords:** lateral geniculate nucleus, Pax6, prethalamus, thalamus, Zic4

## Abstract

Our aim was to study the mechanisms that contribute to the development of discrete thalamic nuclei during mouse embryogenesis (both sexes included). We characterized the expression of the transcription factor coding gene *Zic4* and the distribution of cells that expressed *Zic4* in their lineage. We used genetic fate mapping to show that *Zic4*-lineage cells mainly contribute to a subset of thalamic nuclei, in particular the lateral geniculate nuclei (LGNs), which are crucial components of the visual pathway. We observed that almost all *Zic4*-lineage diencephalic progenitors express the transcription factor Pax6 at variable location-dependent levels. We used conditional mutagenesis to delete either one or both copies of *Pax6* from *Zic4*-lineage cells. We found that *Zic4*-lineage cells carrying either homozygous or heterozygous loss of *Pax6* contributed in abnormally high numbers to one or both of the main lateral geniculate nuclei (LGNs). This could not be attributed to a change in cell production and was likely due to altered sorting of thalamic cells. Our results indicate that positional information encoded by the levels of Pax6 in diencephalic progenitors is an important determinant of the eventual locations of their daughter cells.

## Significance Statement

The development of the thalamus is a process in which cells that initially appear similar give rise to distinct cell groups called nuclei. How these nuclei form is poorly understood. We utilized a mouse model in which cells that express the gene *Zic4* can be followed. We studied the consequences of knocking out either one or both copies of the gene encoding the Pax6 transcription factor in these *Zic4*-lineage cells. We found that these mutations had significant effects on the contribution of *Zic4*-lineage cells to specifically visual thalamic nuclei. This was not attributable to a change in *Zic4*-lineage cell production in mutants. Rather, we suggest that mutation of Pax6 affects the distribution of *Zic4*-lineage neurons to specific thalamic nuclei.

## Introduction

The diencephalon is one of the two major components of the vertebrate forebrain. It contains several structures essential for brain function, including the thalamus and prethalamus. The thalamus is an important regulator of fundamental processes including sleep, alertness, consciousness and cognition, and is involved in the regulation of corticocortical communication and the relaying of sensory information to the cerebral cortex (Sherman and Guillery, 2002, 2006, 2011; Jones 2007). The thalamus is commonly subdivided into >40 distinct nuclei, distinguished according to their function, cytoarchitecture, anatomic connectivity and gene expression patterns (Jones, 2007; Lim and Golden, 2007; Martinez-Ferre and Martinez, 2012). For example, the lateral geniculate nucleus (LGN) is essential for the processing of visual information. The LGN is divided into two major components: the dorsal LGN (dLGN) relays visual signals from the retina to the visual cortex; the ventral LGN (vLGN) is involved in processing and integration, having inputs from retina, cortex, and superior colliculus and connections to other thalamic nuclei.

Thalamic nuclei develop in the mouse embryo during the final third of gestation as neurons generated from progenitors lining the third ventricle migrate radially into the thalamic mantle zone (Nakagawa and Shimogori, 2012). This process is likely to be regulated by the transcription factor Pax6, which is expressed by most diencephalic progenitors. The relatively few exceptions are located at the zona limitans intrathalamica (ZLI) between the thalamus and prethalamus (Macdonald et al., 1995; Ericson et al., 1997; Robertshaw et al., 2013; Caballero et al., 2014). The expression level of Pax6 varies systematically across the thalamus from high (caudally) to low (rostrally) and is high in the prethalamus. It can, therefore, confer positional identity on diencephalic progenitors, which might contribute to the ability of their daughter neurons to coalesce into discrete nuclei later in development.

Zic4 is a zinc finger transcription factor expressed in embryonic mouse nervous system, including the diencephalon, from embryonic day (E)9.5 on (Aruga et al., 1996a,b; Gaston-Massuet et al., 2005). A previous study reported that it is highly expressed in the postnatal LGN (Horng et al., 2009). We began our study by testing whether cells related by the expression of *Zic4* at some time in their lineage (referred to as *Zic4*-lineage cells) contribute selectively to specific thalamic nuclei. We conducted a detailed analysis of the development of both *Zic4* expression and the distributions of *Zic4*-lineage cells in the embryonic diencephalon and found that *Zic4*-lineage cells are distributed preferentially to a select subset of thalamic nuclei, in particular the vLGN, by the time of birth. We then studied the consequences of *Zic4^Cre^* induced deletions of either one or both copies of *Pax6*.

## Materials and Methods

### Mice

All experiments were conducted in accordance with Home Office United Kingdom regulations and University of Edinburgh animal welfare guidelines.

Conditional *Pax6* knock-out mice were generated by crossing floxed *Pax6* mutant mice *Pax6^fl/fl^*(Simpson et al., 2009) with *Zic4^Cre^* mice (Rubin et al., 2010), a kind gift from Dr. Thomas Theil at Center for Discovery Brain Sciences at the University of Edinburgh. These mice were crossed with *RCE:loxP* mice (Miyoshi et al., 2010) to report the Cre activity with the expression of an enhanced green fluorescent protein (EGFP; subsequently referred to as GFP). The triple transgenic mice were maintained on a C57BL/6 background. To obtain *Zic4^Cre+/−^;Pax6^fl/fl^;RCE*^+/−^, *Zic4^Cre+/−^;Pax6^fl/+^;RCE*^+/−^, and *Zic4^Cre+/−^;Pax6^+/+^*;*RCE*^+/−^ embryos (subsequently referred to as *Pax6^fl/fl^*, *Pax6^fl/+^*, and *Pax6^+/+^*), *Pax6^fl/+^;RCE*^+/−^ females were crossed with *Pax6^fl/+^;Zic4^Cre+/−^* males. To obtain *Zic4^Cre+/−^;RCE*^+/−^ embryos, *RCE*^+/−^ females were crossed with *Zic4^Cre+/−^* males.

### Genotyping

*Zic4^Cre+/−^* mice were genotyped using primers (forward: GAGGGACTACCTCCTGTACC, reverse: TGCCCAGAGTCATCCTTGGC) to the *iCre* cassette (Rubin et al., 2010), resulting in a 630-bp PCR product in the mutant. *RCE*^+/−^ mice were genotyped using three primers (Rosa1: CCCAAAGTCGCTCTGAGTTGTTATC; Rosa2: GAAGGAGCGGGAGAAATGGATATG; and Cag3: CCAGGCGGGCCATTTACCGTAAG) to the EGFP reporter (Miyoshi et al., 2010), resulting in a 550-bp PCR product in the wild type and a 350-bp PCR product in the mutant. Mice carrying the *Pax6^fl^* transgene (Simpson et al., 2009) were genotyped using two primers (FP6GtF: AAATGGGGGTGAAGTGTGAG; FP6GtR: TGCATGTTGCCTGAAAGAAG), resulting a 156-bp PCR product in the wild type and a 195-bp PCR product in the mutant.

All *Zic4^Cre+/−^;RCE*^+/−^ embryos and pups were distinguished from wild-type mice based on the detection of the GFP reporter using a GFP stereoscope and genotyped for the *Pax6^fl^* transgene using the primers described above.

### Tissue preparation and histology

The day that the vaginal plug was found was considered as E0.5. For postnatal studies, the birth date was considered as postnatal day (P)0. Embryos were extracted from pregnant females by caesarean section following an overdose of isoflurane and were harvested into ice-cold PBS. For embryos aged from E11.5 to E14.5, whole heads were removed and fixed in 4% (w/v) paraformaldehyde (PFA) in PBS overnight at 4°C. For embryos aged from E15.5 to E18.5, brains were dissected from skulls before proceeding to fixation. P0 pups were anaesthetized by an overdose of sodium pentobarbitone and perfused with 4% PFA through the left ventricle of the heart. The brains were extracted and immersed in 4% PFA for 48 h at 4°C.

Fixed samples were either dehydrated in 70% EtOH and embedded in paraffin for microtome sectioning or cryo-protected in 30% sucrose (w/v) in PBS, embedded in a 1:1 mixture of 30% sucrose (w/v) in PBS and optimal cutting temperature (OCT) compound (Tissue-Tek, Sakura Finetek Europe), and frozen on dry ice for cryostat sectioning. All samples were sectioned coronally at 10 μm.

### Fluorescent *in situ* hybridization

The digoxigenin (DIG)-labeled *Zic4* antisense RNA probe was used at a concentration of 1:2000. Fluorescent *in situ* hybridizations were performed according to a published protocol (Rubin et al., 2010). The plasmid used to generate the *Zic4* probe was the IMAGE clone 6400880 (linearised with EcoRI and transcribed with T3 RNA polymerase), a kind gift from Professor Nicoletta Kessaris, Wolfson Institute for Biomedical Research, University College London, United Kingdom.

### Immunohistochemistry

Fluorescent immunohistochemistry was performed on paraffin embedded sections according to previously published protocols (Martynoga et al., 2005). Diaminobenzidine (DAB) immunohistochemistry for Pax6 was performed using the Vectastain ABC kit (Vector Laboratories) following an incubation of slides in biotinylated secondary antibodies and slides were incubated in 3% hydrogen peroxide/90% methanol for 30 min to inactivate endogenous peroxidase activities during the rehydration process.

Primary antibodies used were as follow: mouse anti-BrdU IgG1 (1:150, BD Biosciences, RRID:AB_10015219), rabbit anti-Ki67 IgG (1:500, Abcam, RRID:AB_2049848), rabbit anti-pHH3 (Ser10; 1:100, Millipore), mouse anti-Tuj1 IgG (1:400, Abcam), goat anti-GFP IgG (1:150, Abcam), mouse anti-COUP-TFI IgG_2A_ (1:500, R&D Systems, RRID:AB_1964211), mouse anti-COUP-TFII IgG_2A_ (1:1000, R&D Systems, RRID:AB_1964214), mouse anti-LIM1/2 [1:50, Developmental Studies Hybridoma Bank (DSHB), University of Iowa], mouse anti-Nkx2.2 (1:200, DSHB), and mouse anti-Pax6 (AD2.38; a kind gift from Professor Veronica Van Heyningen at Institute of Genetics and Molecular Medicine, University of Edinburgh).

Secondary antibodies used were donkey anti-goat Alexa Fluor 488 (1:200, Invitrogen), rabbit anti-mouse biotin (1:200, Vector Laboratories), goat anti-rabbit biotin (1:200, Vector Laboratories), streptavidin Alexa Fluor 594 (1:200, Invitrogen), donkey anti-mouse Alexa Fluor 568 (1:200, Invitrogen), donkey anti-mouse Alexa Fluor 647 (1:200, Invitrogen), and donkey anti-rabbit Alexa Fluor 647 (1:200, Invitrogen).

### Bromodeoxyuridine (BrdU) labeling

A single dose of BrdU (70 μg/g mouse weight, diluted in saline 10 μg/μl) was administrated via intraperitoneal injection to pregnant dams carrying E10.5, E11.5, E12.5, and E13.5 embryos. For birthdate studies, brain tissue was collected from four P0 *Zic4Cre*^+/–^*;RCE*^+/–^*;Pax6^+/+^* pups for each injection age (E10.5–E13.5). For cell proliferation studies, four embryos were collected for brain tissue 1.5 h after injection for each injection age (E11.5–E12.5).

### Imaging, image processing, and cell counting

DAB images were taken with a Leica DFC480 camera connected to a Leica DMNB epifluorescence microscope. Fluorescence images were taken with a Leica DM5500B automated upright microscope connected to a DFC360FX camera and a Nikon A1R confocal microscope.

For birthdating studies, vLGN, dLGN, and ventral posterior (VP) nuclei were outlined and processed in CellProfiler (Lamprecht et al., 2007) using a custom pipeline. RGB images were split into three single-channel gray scale images and a global thresholding was performed to reduce background noises. Cell nuclei were identified using DAPI staining and a cutoff intensity value of 5 was used to distinguish nuclei with positive or negative staining for both BrdU and GFP. All cell nuclei were classified and automatically counted into four categories: GFP-BrdU-, GFP-BrdU+, GFP+BrdU+, and GFP+BrdU-, and proportions of each category were calculated and exported into spreadsheets.

For cell proliferation studies, a box with a fixed width was positioned and stretched to include the whole diencephalic wall for three structures of interest: the prethalamus and the pTH-R and pTH-C in the thalamus. Cropped images were processed in CellProfiler. Cell nuclei segmentation and fluorescence intensity measurements for BrdU, pHH3, Ki67, Tuj1, and GFP were performed as described above, with the same intensity cutoff applied ahead of cell counting.

For cell contribution studies, volumes of vLGN, dLGN, and VP were estimated using the ImageJ Volumest plugin (Merzin, 2008) using a series of 10 coronal sections with a regular interval of 60 μm. Within each section, nuclei were outlined and overlaid with a counting grid. 30% of counting tiles (30 × 30 μm) were randomly sampled using a custom ImageJ plugin (inspired by Wayne Rasband’s ImageJ plugin Grid) and manually counted to calculate densities of all cells and GFP+ cells. The number of all cells in each nucleus was calculated by multiplying its volume and cell density.

### Code accessibility

Custom CellProfiler pipeline and ImageJ sampling tile generator plugin will be provided on request.

### Statistical analyses

Statistical analyses were conducted using the Prism 7 statistical software (version 7.01, GraphPad Software Inc.). Univariate statistics (mean ± SEM) were performed for all studied variables. One-way ANOVA was performed to study the effect of genotype, and two-way ANOVA was performed to examine the effects of genotype and anterior-posterior position. The Tukey’s *post hoc* test was performed for all pair-wise comparisons subsequent to the ANOVAs.

## Results

### *Zic4* and *Zic4^Cre^* expression in the embryonic diencephalon

**Figure 1.**
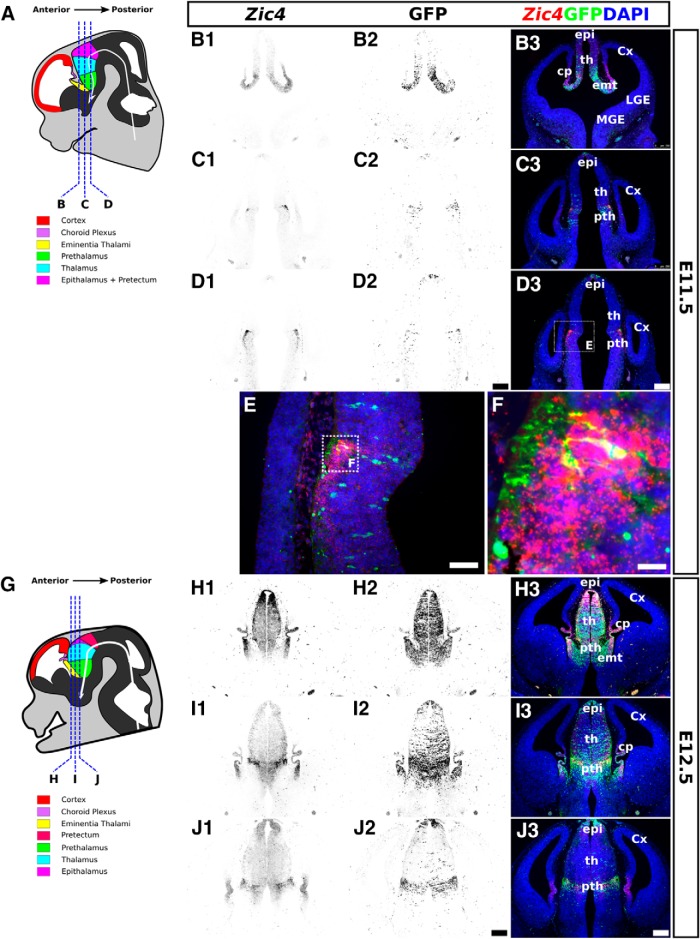
Expression of *Zic4* and activity of *Zic4^Cre^* in *Zic4^Cre+/−^*;*RCE^+/−^* mouse brain coronal sections at E11.5 and E12.5. ***A***, ***G***, Schematics of E11.5 and E12.5 mouse brain demarcating major subdivisions and sectioning planes. ***B1–F***, ***H1–J3***, *Zic4* fluorescent *in situ* hybridization (monochrome in ***B1***, ***C1***, ***D1***, ***H1***, ***I1***, ***J1*** or red in ***B3***, ***C3***, ***D3***, ***E***, ***F***, ***H3***, ***I3***, ***J3***) and immunohistochemistry for GFP reporter of *Zic4^Cre^* activity (monochrome in ***B2***, ***C2***, ***D2***, ***H2***, ***I2***, ***J2*** or green in ***B3***, ***C3***, ***D3***, ***E***, ***F***, ***H3***, ***I3***, ***J3***) with DAPI counterstaining (blue). Box in ***D3*** is shown in ***E***; box in ***E*** is shown in ***F***. Scale bars: 250 µm (***B1–D3***, ***H1–J3***), 125 µm (***E***), 25 µm (***F***). Cx, cortex; cp, choroid plexus; MGE, medial ganglionic eminence; LGE, lateral ganglionic eminence; th, thalamus; pth, prethalamus; emt, eminentia thalami; epi, epithalamus.

**Figure 2.**
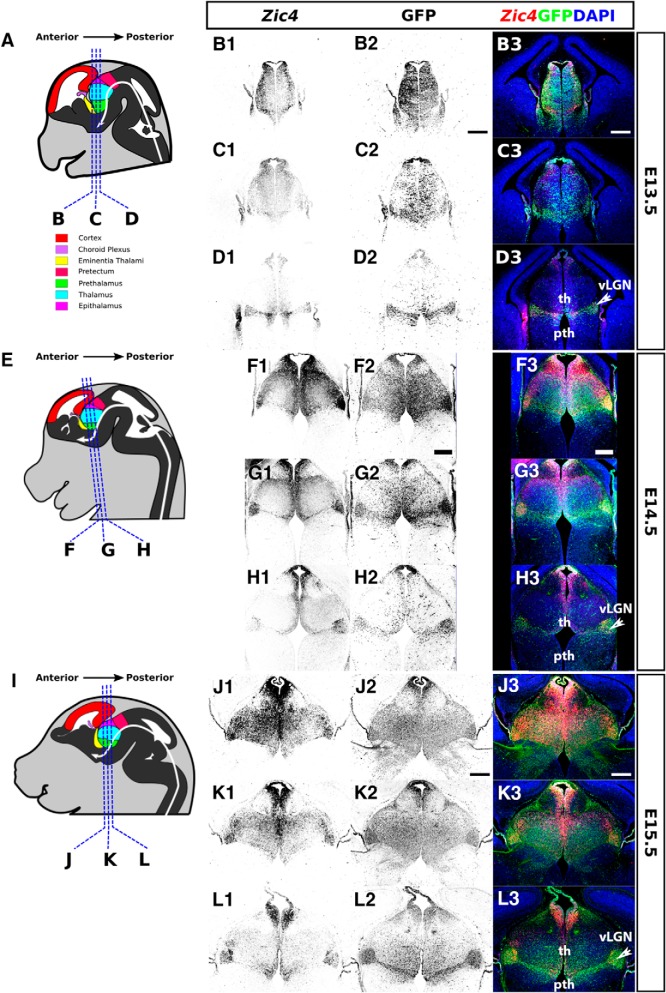
Expression of *Zic4* and activity of *Zic4^Cre^* in *Zic4^Cre+/−^*;*RCE^+/−^* mouse brain coronal sections at E13.5, E14.5, and E15.5. ***A***, ***E***, ***I***, Schematics of E13.5, E14.5, and E15.5 mouse brain demarcating major subdivisions and sectioning planes. ***B1–D3***, ***F1–H3***, ***J1–L3***, *Zic4* fluorescent *in situ* hybridization (monochrome in ***B1***, ***C1***, ***D1***, ***F1***, ***G1***, ***H1***, ***J1***, ***K1***, ***L1*** or red in ***B3***, ***C3***, ***D3***, ***F3***, ***G3***, ***H3***, ***J3***, ***K3***, ***L3***) and immunohistochemistry for GFP reporter of *Zic4^Cre^* activity (monochrome in ***B2***, ***C2***, ***D2***, ***F2***, ***G2***, ***H2***, ***J2***, ***K2***, ***L2*** or green in ***B3***, ***C3***, ***D3***, ***F3***, ***G3***, ***H3***, ***J3***, ***K3***, ***L3***) with DAPI counterstaining (blue). Scale bars: 250 µm. vLGN, ventral lateral geniculate nucleus; th, thalamus; pth, prethalamus.

**Figure 3.**
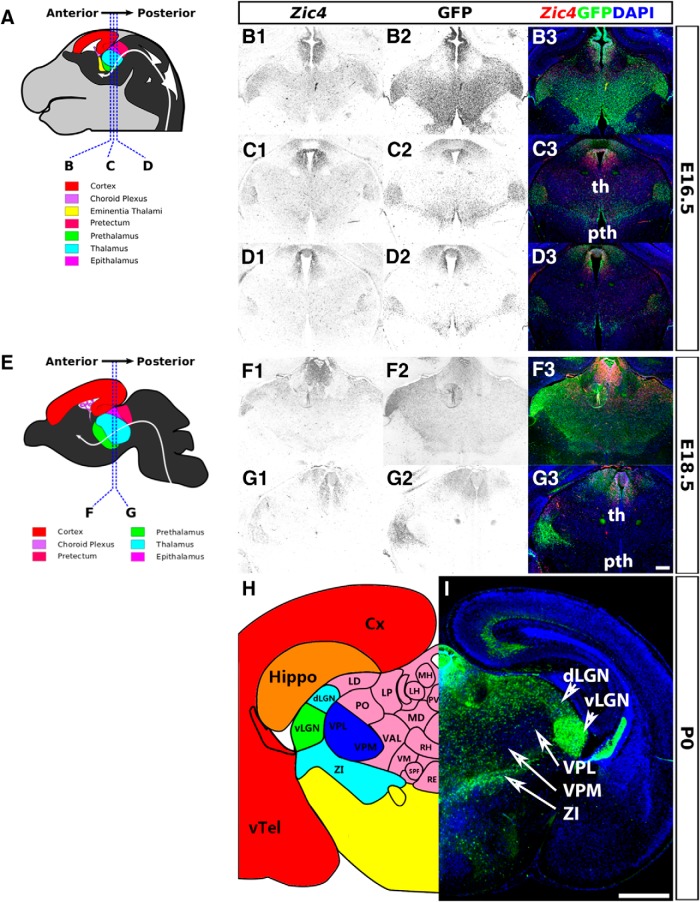
Expression of *Zic4* and activity of *Zic4^Cre^* in *Zic4^Cre+/−^*;*RCE^+/−^* mouse brain coronal sections at E16.5, E18.5, and P0. ***A***, ***E***, ***H***, Schematics of E16.5, E18.5, and P0 mouse brain demarcating major subdivisions and nuclei and sectioning planes. ***B1–D3***, ***F1–G3***, *Zic4* fluorescent *in situ* hybridization (monochrome in ***B1***, ***C1***, ***D1***, ***F1***, ***G1*** or red in ***B3***, ***C3***, ***D3***, ***F3***, ***G3***) and immunohistochemistry for GFP reporter of *Zic4^Cre^* activity (monochrome in ***B2***, ***C2***, ***D2***, ***F2***, ***G2*** or green in ***B3***, ***C3***, ***D3***, ***F3***, ***G3***, ***I***) with DAPI counterstaining (blue). Scale bars: 250 µm (***B1–G3***), 500 µm (***I***). Cx, cortex; dLGN, dorsal lateral geniculate nucleus; Hippo, hippocampus; LD, lateral dorsal nucleus; LH, lateral habenula; LP, lateral posterior nucleus; MD, medial dorsal nucleus; MH, medial habenula; PO, posterior nucleus; pth, prethalamus; PV, paraventricular nucleus; RE, nucleus reunions; RH, rhomboid nucleus; SPF, subparafascicular thalamic nucleus; th, thalamus; VAL, ventral anterolateral nucleus; vLGN, ventral lateral geniculate nucleus; VM, ventromedial nucleus; VPL, ventral posterolateral nucleus; VPM = ventral posteromedial nucleus; vTel, ventral telencephalon; ZI, zona incerta. ***H***, Adapted from Allen Brain Atlas (http://developingmouse.brain-map.org/).

We began by thoroughly analyzing and comparing the expression of *Zic4* transcripts and GFP reporter in the *Zic4Cre*^+/–^;*RCE*^+/–^ diencephalon at embryonic stages from E11.5 to birth (Figs. 1–3). Whereas *in situ* hybridizations for *Zic4* mRNA revealed the expression patterns of the gene at the time of analysis, GFP revealed cells that were either expressing or at some time in their past expressed *Zic4* (*Zic4*-lineage cells).

In the E11.5 diencephalon (Fig. 1*A–F*), *Zic4* transcripts were detected at highest levels in cells of the prethalamus (Fig. 1*C1*,*D1*) and the eminentia thalami (Fig. 1*B1*). In the prethalamus, the *Zic4* transcripts were concentrated in cells on the outer, pial side of the neuroepithelium (Fig. 1*E*). GFP expression overlapped that of *Zic4* transcripts, with GFP+ cells most frequent in the prethalamus and the eminentia thalami (Fig. 1*B2*,*C2*,*D2*). GFP reporter activity did not correspond perfectly to the expression of *Zic4* transcripts, with many *Zic4*+ cells being GFP negative (Fig. 1*E*,*F*). The most likely explanation for this was that insufficient time had elapsed following the onset of *Zic4* expression for the production of detectable levels of GFP. This delay would have been for the production of Cre recombinase from the *Zic4Cre* transgene, the consequent activation of the reporter gene and the production of sufficient GFP.

At E12.5, *Zic4* transcripts were present more widely, from epithalamus through thalamus and prethalamus to eminentia thalami (Fig. 1*H1–J1*). Expression levels were still highest in the prethalamus, as at E11.5, but were also strong in the epithalamus. In the thalamus, expression levels were highest in more anterior sections. GFP expression showed very similar patterns (Fig. 1*H2–J2*).

These E12.5 patterns of *Zic4* and GFP expression were largely maintained as the tissues grew in size over subsequent days up to birth (Figs. 2, 3). As thalamic nuclei formed during this period, *Zic4*/GFP+ cells became concentrated in the vLGN, around the border between the thalamus and prethalamus, in anterior thalamic regions and in the epithalamus. By birth, the densest concentration of GFP+ *Zic4*-lineage cells was in vLGN and the medial habenula of the epithalamus (Fig. 3*H*,*I*). Their densities were intermediate in other lateral thalamic nuclei such as the dLGN and in the zona incerta (ZI) of the prethalamus. They were low in midline thalamic nuclei such as nucleus reuniens and the rhomboid nucleus, and almost absent from VP thalamic nuclei such as the ventral posterolateral nucleus (VPL) and ventral posteromedial nucleus (VPM; Fig. 3*H*,*I*).

These findings indicate that *Zic4*-lineage diencephalic progenitors contribute their daughter cells mainly to the thalamic nuclei that are close to the boundary between thalamus and prethalamus, particularly to the vLGN but also to others such as the dLGN.

### Overlap between *Zic4*-lineage and Pax6-expressing cells

**Figure 4.**
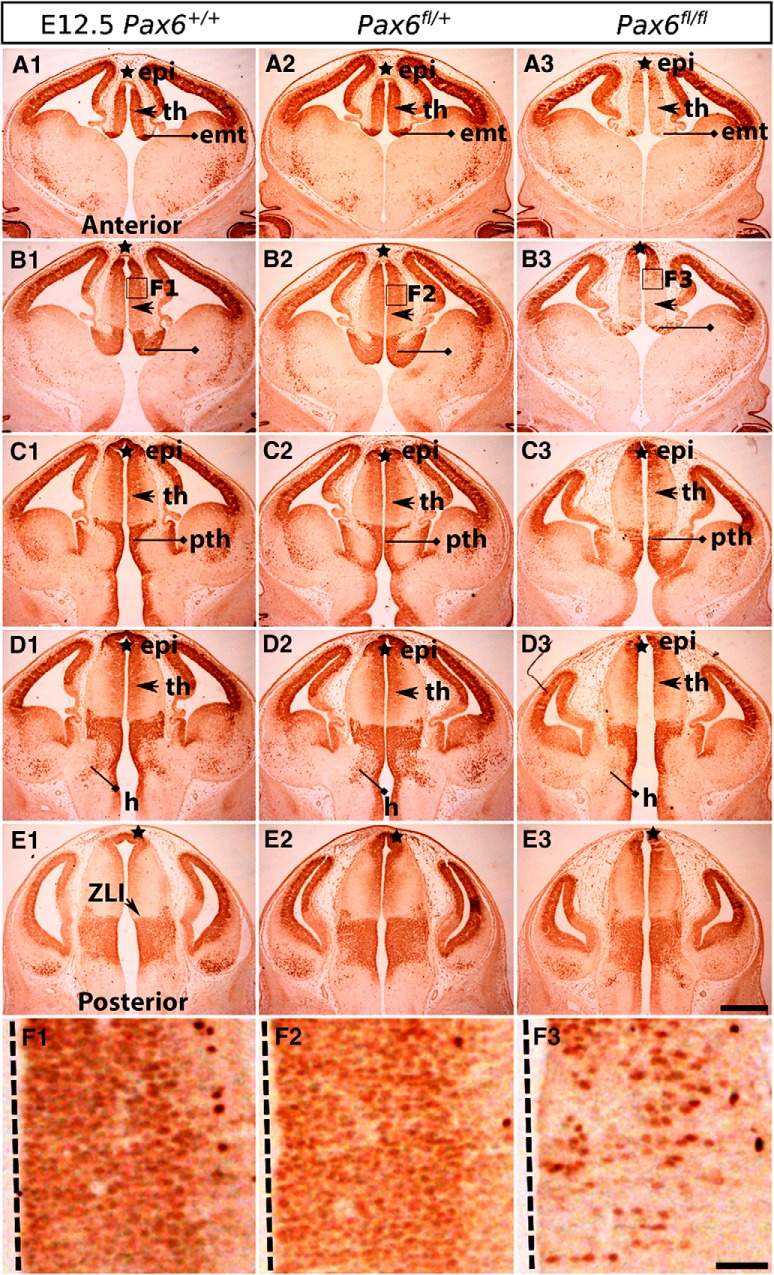
Pax6 expression in control and Pax6 mutant embryos at E12.5. Immunohistochemistry for Pax6 on a set of sections cut through the diencephalon in similar planes of section to those shown in Figure 1. Boxed areas in ***B1–B3*** are enlarged in ***F1–F3***. ZLI, zona limitans intrathalamica; epi, epithalamus; th, thalamus; pth, prethalamus; emt, eminentia thalami; h, hypothalamus. Scale bars: 500 µm (***A1–E3***), 50 µm (***F1–F3***).

The normal expression pattern of Pax6 is shown at E12.5, E13.5, E14.5, and E16.5 in Figures 4*A1–F1*, 5*A1–G1*, 6*A1–G1*, 7*A1–F1*. Pax6 is expressed at some level in almost all progenitors throughout the epithalamus, thalamus, prethalamus, and eminentia thalami, the exceptions being located in the ZLI, a small strip of tissue between the thalamus and prethalamus (Fig. 4*E1*). In thalamus, it is distributed in a gradient with the lowest levels close to the border with the prethalamus (Figs. 4*A1–E1*, 5*A1–E1*). Since it is not expressed by postmitotic thalamic neurons, it almost completely disappears once the progenitor population is exhausted by E16.5 (Fig. 7*A1–F1*).

**Figure 5.**
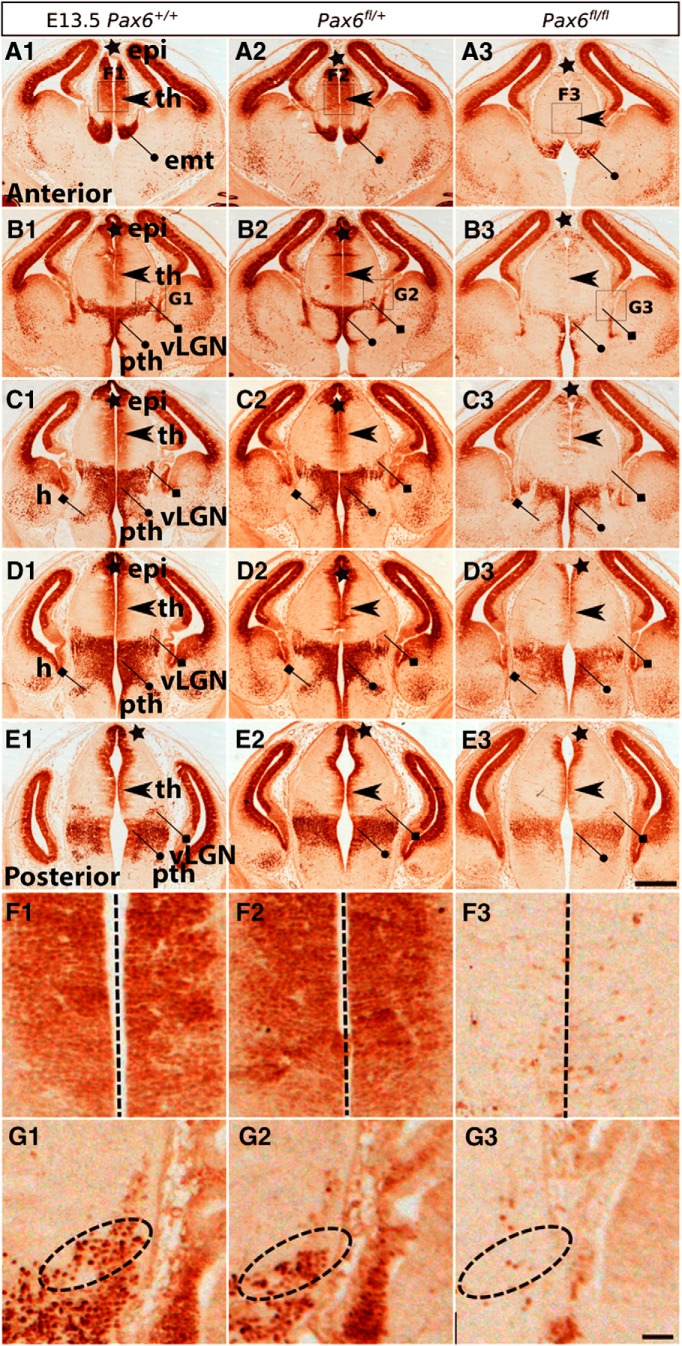
Pax6 expression in control and Pax6 mutant embryos at E13.5. Immunohistochemistry for Pax6 on a set of sections cut through the diencephalon in similar planes of section to those shown in Figure 2. Boxed areas in ***A1–B3*** are enlarged in ***F1–G3***. vLGN, ventral lateral geniculate nucleus; epi, epithalamus; th, thalamus; pth, prethalamus; emt, eminentia thalami; h, hypothalamus. Scale bars: 500 µm (***A1–E3***), 50 µm (***F1–F3***).

**Figure 6.**
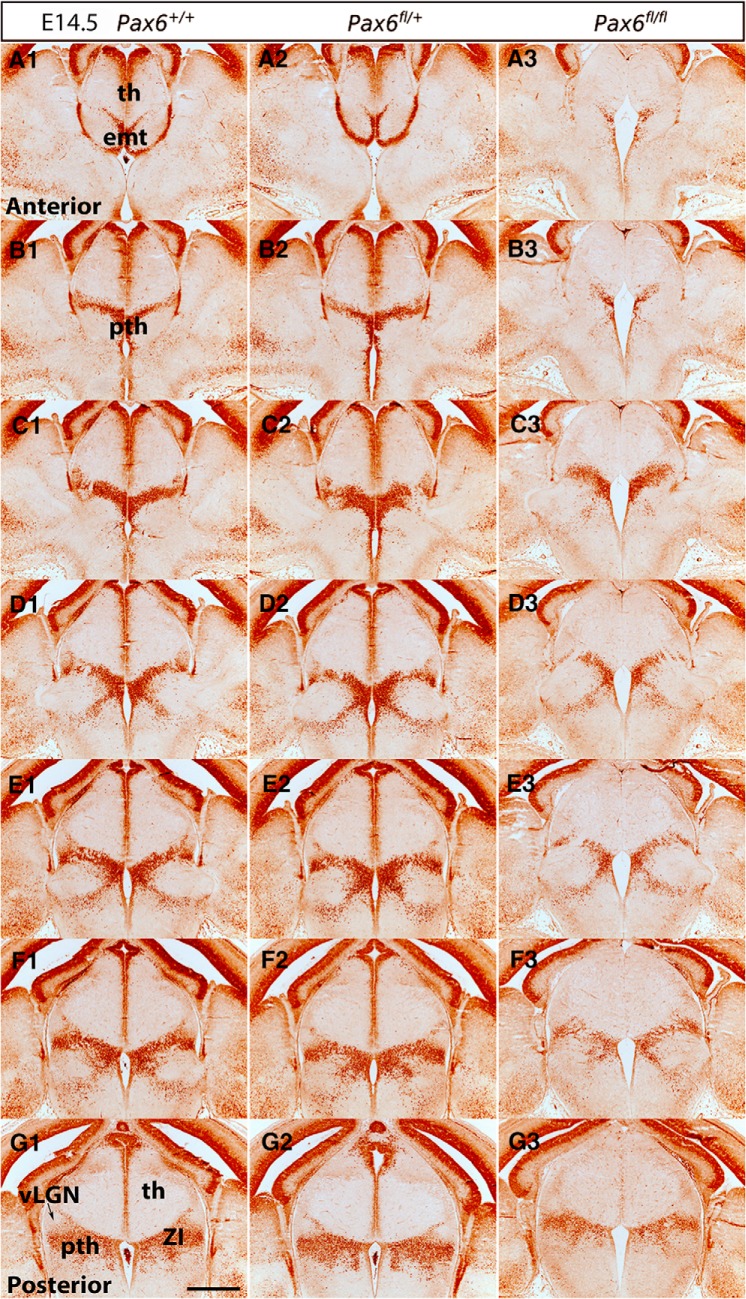
Pax6 expression in control and Pax6 mutant embryos at E14.5. (***A1–G3***) Immunohistochemistry for Pax6 on a set of sections cut through the diencephalon in similar planes of section to those shown in Figure 2, arranged from anterior (***A1–A3***) to posterior (***G1–G3***). ZI, zona incerta; vLGN, ventral lateral geniculate nucleus; th, thalamus; pth, prethalamus; emt, eminentia thalami. Scale bar: 500 µm.

**Figure 7.**
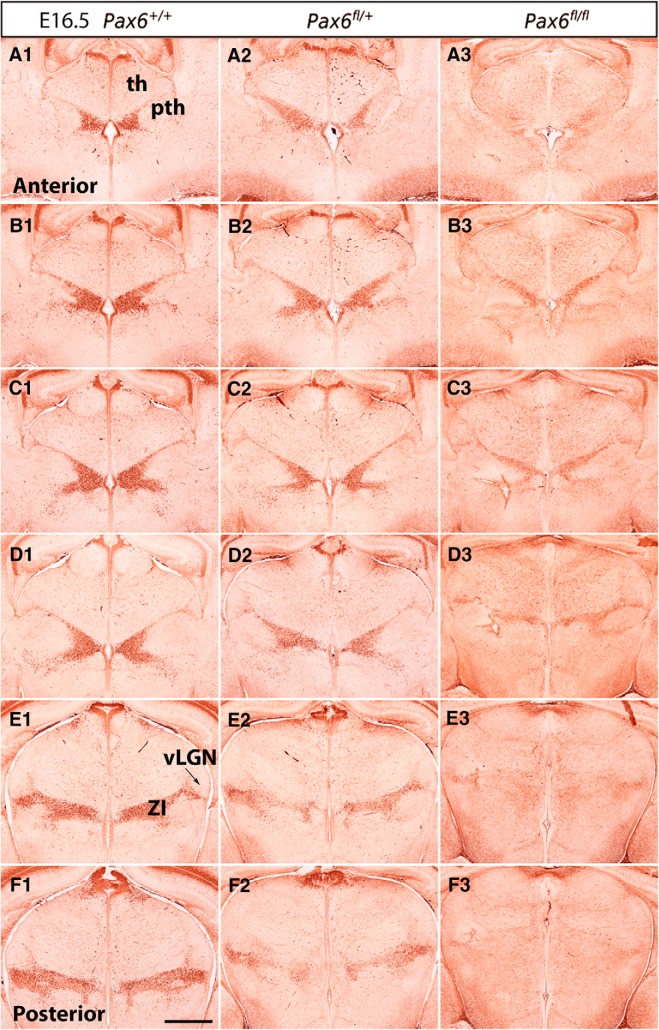
Pax6 expression in control and Pax6 mutant embryos at E16.5. (***A1–F3***)Immunohistochemistry for Pax6 on a set of sections cut through the diencephalon in similar planes of section to those shown in Figure 3, arranged from anterior (***A1–A3***) to posterior (***F1–F3***). ZI, zona incerta; vLGN, ventral lateral geniculate nucleus; th, thalamus; pth, prethalamus. Scale bar: 500 µm.

In the prethalamus, Pax6 is strongly expressed by progenitors and is retained by many postmitotic cells as they migrate radially into the diencephalic mantle zone (Figs. 4*A1–E1*, 5*A1–E1*, 6*A1–G1*). These cells form a Pax6-positive strip running across the prethalamus. At early stages, some of them migrate as far as the outer edge of the neuroepithelium to a region where the vLGN will form (Fig. 5*B1–E1*,*G1*). By E14.5, when the development of discrete diencephalic nuclei is underway, most Pax6-expressing cells are located in the ZI of the prethalamus (Fig. 6*A1–G1*). At E14.5 and E16.5, we observed small numbers of Pax6-expressing cells in the vicinity of the vLGN, mostly around rather than within it (Figs. 6*E1–G1*, 7*D1–F1*).

**Figure 8.**
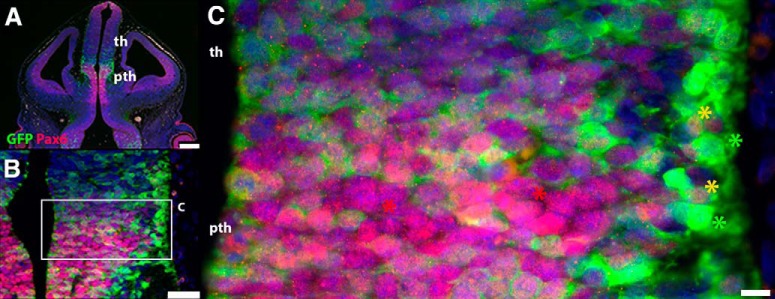
Diencephalic *Zic4*-lineage cells express Pax6. Double-immunohistochemistry for GFP (*Zic4*-lineage cells) and Pax6 at E11.5. Area outlined in ***B*** is enlarged in ***C***. Asterisks in ***C***: yellow, examples of double-labeled cells; green, examples of cells labeled only with GFP; red, examples of cells labeled only for Pax6. Scale bars: 250 µm (***A***), 50 µm (***B***), 10 µm (***C***).

As expected, given that Pax6 is expressed by almost all thalamic and prethalamic progenitors, double-label experiments showed that *Zic4*-lineage thalamic and prethalamic progenitor cells express Pax6 as early as E11.5 (Fig. 8). Many *Zic4*-lineage postmitotic cells in the prethalamus retained Pax6 (e.g., yellow asterisks in Fig. 8*C*); others did not (e.g., green asterisks in Fig. 8*C*). We went on to use the *Zic4^Cre^* allele to test the effects of mutating one or both copies of the *Pax6* gene in *Zic4*-lineage cells.

### *Zic4^Cre^*-induced deletion of Pax6

We studied Pax6 expression in *Zic4^Cre^*;*Pax6^fl/+^* and *Zic4^Cre^*;*Pax6^fl/fl^* embryos at E12.5, E13.5, E14.5, and E16.5. For each genotype at each age, we examined three non-littermate embryos and the results were indistinguishable. In *Zic4^Cre^*;*Pax6^fl/fl^* homozygous mutant embryos (Figs. 4*A3–F3*, 5*A3–G3*, 6*A3–G3*, 7*A3–F3*), the numbers of cells immunostained for Pax6 declined rapidly in the thalamus from E12.5 onwards, particularly in its anterior parts where many cells are *Zic4*-lineage. By E14.5, there were almost no Pax6-expressing progenitors in the thalamus in *Zic4^Cre^*;*Pax6^fl/fl^* embryos whereas normally a small population remained. The numbers of Pax6-expressing cells in the prethalamus of *Zic4^Cre^*;*Pax6^fl/fl^* embryos were greatly reduced at all ages. There were no consistent defects in the overall shapes and sizes of diencephalic structures.

In *Zic4^Cre^*;*Pax6^fl/+^* heterozygotes, we detected no defects of Pax6 expression at E12.5–E14.5 (Figs. 4*A2–F2*, 5*A2–G2*, 6*A2–G2*). By E16.5, however, the numbers of Pax6-expressing cells had declined throughout the prethalamus and the intensity of the immunostaining of many Pax6-expressing cells was lower than normal (Fig. 7*A2–F2*). The pattern of Pax6 expression in *Zic4^Cre^*;*Pax6^fl/+^* embryos now appeared intermediate between controls and *Zic4^Cre^*;*Pax6^fl/fl^* embryos. There were no detectable defects in the positions of the Pax6-expressing cells. The overall shapes and sizes of diencephalic structures appeared normal. These findings indicate that mutation of one copy of *Pax6* in *Zic4*-lineage cells causes them to lower their Pax6 levels between E14.5 and E16.5, i.e., once they are postmitotic neurons, with levels in some falling below the threshold for detection.

### Cells of the vLGN, dLGN, and the VP nuclei are generated before E14.5

We selected three thalamic nuclei to search for defects resulting from mutation of *Pax6* in *Zic4*-lineage cells: vLGN, which contains a large proportion of *Zic4*-lineage cells at birth; dLGN, which contains an intermediate proportion of *Zic4*-lineage cells at birth; VP nuclei (VPM and VPL), which contain low numbers of *Zic4*-lineage cells at birth.

**Figure 9.**
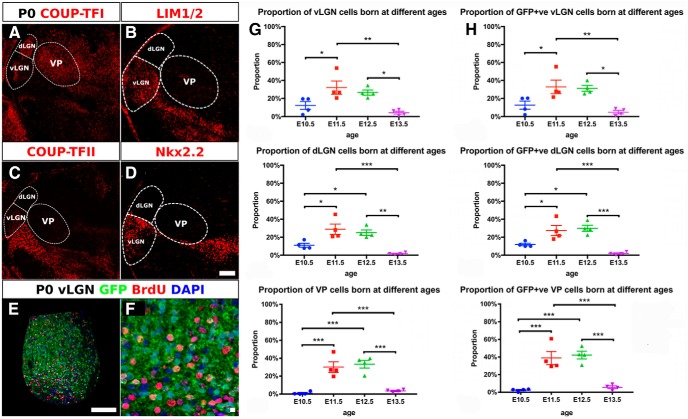
Birthdates of cells in the vLGN, dLGN, and VP. ***A–D***, Immunohistochemistry at P0 with markers designed to help delineate borders of the thalamic nuclei analyzed here. Scale bar: 100 µm. ***E***, ***F***, Example of BrdU and GFP immunohistochemistry in vLGN. Scale bars: 100 µm (***E***), 10 µm (***F***). ***G***, Proportions of all cells in each nucleus that were BrdU+ after injection with BrdU at E10.5–E13.5. ***H***, Proportions of GFP-expressing cells in each nucleus that were BrdU+ after injection with BrdU at E10.5–E13.5. Data points are from individual animals. Mean ± SEM are shown in each case. One-way ANOVA returned significant effects of age in all cases: (***G***) vLGN *F*_(3,12)_ = 7.729, *p* = 0.0039; dLGN *F*_(3,12)_ = 13.56, *p* = 0.0004; VP *F*_(3,12)_ = 20.76, *p* < 0.0001; (***H***) vLGN *F*_(3,12)_ = 8.282, *p* = 0.0030; dLGN *F*_(3,12)_ = 15.41, *p* = 0.0002; VP *F*_(3,12)_ = 23.26, *p* < 0.0001. Holm–Sidak’s multiple comparisons tests were performed following one-way ANOVA: *p < 0.05; **p < 0.01; ***p < 0.001.

As a first step toward testing whether *Pax6* mutation in *Zic4*-lineage cells affects the progenitors that give rise to these nuclei, we checked the ages at which vLGN, dLGN, and VP cells are generated. We injected BrdU into control embryos at E10.5–E13.5 and studied BrdU labeling at birth (P0). To help identify the nuclei we used several markers: COUP-TFI, COUP-TFII, LIM1/2, and Nkx2.2 (Fig. 9*A–D*). COUP-TFI is expressed across most of the thalamic nuclei including the vLGN, dLGN, and VP. The expression level of COUP-TFI in the vLGN is lower than the surrounding nuclei such as the dLGN and VP and, within the vLGN, is highest in the middle of the nucleus (Fig. 9*A*). COUP-TFII is expressed strongly in the prethalamus and helped determine the anterior boundaries of the vLGN and VP (Fig. 9*C*). LIM1/2 and Nkx2.2 are strongly expressed in the vLGN but not in the dLGN or VP (Fig. 9*B*,*D*).

We counted BrdU-labeled cells as a proportion of all cells and as a proportion of *Zic4*-lineage (GFP+) cells in each nucleus (Fig. 9*E*,*F*). We found that the vast majority of cells in vLGN, dLGN, and VP are generated after E10.5 and before E13.5 (Fig. 9*G*,*H*).

### Effects of *Pax6* mutation in *Zic4*-lineage cells on cell production

**Figure 10.**
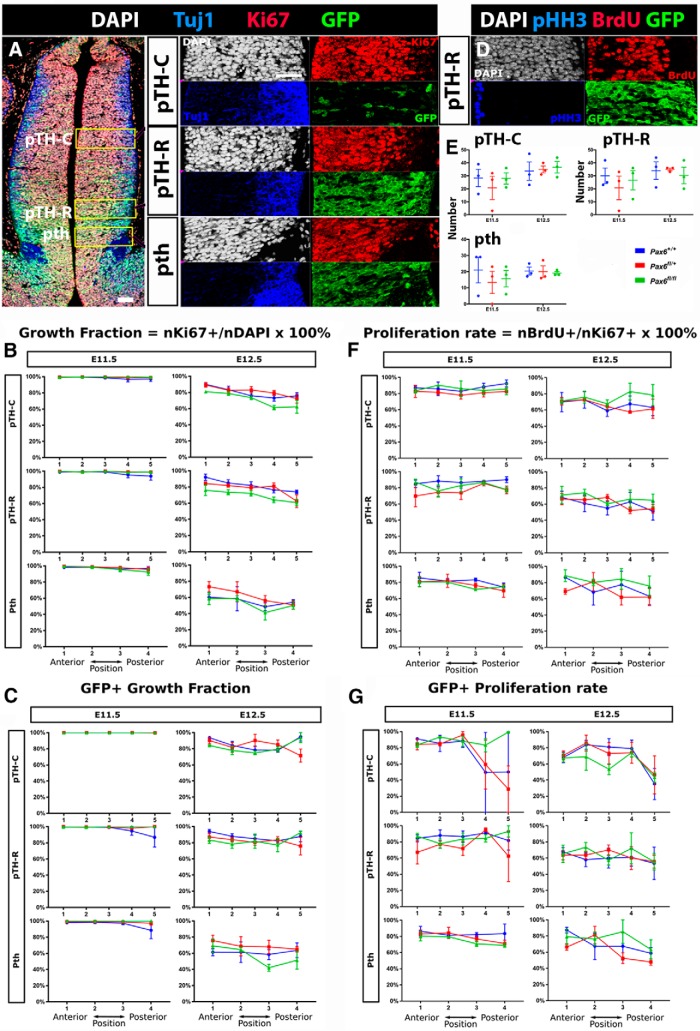
Effects of *Pax6* mutation in *Zic4*-lineage cells on early diencephalic progenitor proliferation. ***A***, Triple immunostaining for Tuj1, Ki67, and GFP. Three regions of interest were selected for analysis, one midway through pTH-C, one in pTH-R and one in prethalamus (pth). Scale bars: 50 µm. ***B***, Average growth fractions (±SEM) in five equally spaced sections through pTH-C and pTH-R and four through prethalamus at E11.5 and E12.5; color coding for genotypes as in ***E***. Two-way ANOVA detected significant effects of genotype only in pTH-C (*F*_(2,30)_ = 9.109, *p* = *0.*0008) and pTH-R (*F*_(2,30)_ = 11.97, *p* = 0.0002) at E12.5 (*n* = 3 embryos of each genotype), with *Pax6^fl/fl^* embryos showing lower growth fractions at all levels (*post hoc* Tukey’s multiple comparisons test, *p* < 0.01 at all positions). ***C***, Average growth fraction for GFP+ cells only. There were no significant effects of genotype. ***D***, An example of triple immunostaining for pHH3, BrdU, and GFP in one region of interest. ***E***, Mean (±SEM) counts of the total numbers of pHH3+ cells in all sampling regions from each domain for each genotype. There were no significant effects of genotype. ***F***, Average proliferation rates (±SEM) in five equally spaced sections through pTH-C and pTH-R and four through prethalamus at E11.5 and E12.5; color coding for genotypes as in ***E***. Two-way ANOVA showed a significant effect of genotype in pTH-R at E11.5 (*F*_(2,30)_ = 4.206, *p* = 0.0245), with lower values in *Pax6^fl/+^* embryos than in the other two genotypes. ***G***, Average proliferation rates for GFP+ cells only. There were no significant effects of genotype.

We next used immunostaining for Ki67 (a marker of proliferating cells), Tuj1 (a marker of newly differentiating neurons) and phosphohistone H3 (pHH3; a marker of cells in M-phase of the cell cycle) together with short-term BrdU labeling to assess the activity of progenitors in regions that generate vLGN, dLGN, and VP at E11.5 and E12.5 (Fig. 10). We made measurements in three regions: pTH-C, which gives rise to thalamic nuclei including dLGN and VP; pTH-R, which contributes to nuclei including vLGN; and prethalamus (Fig. 10*A*). We sampled from four sections through each prethalamus and five through each pTH-C and pTH-R, equally spaced from anterior to posterior, from four embryos of each age and genotype.

We first made measurements on all cells in the three regions, i.e., including both GFP+ and GFP- cells. When we examined the percentages of all cells that were proliferative based on their expression of Ki67, referred to as the growth fraction, we found small but significant decreases in *Pax6^fl/fl^* pTH-C and pTH-R (Fig. 10*B*). When we measured this same parameter specifically in GFP+ (*Zic4*-lineage) cells, we found no significant effects of genotype (Fig. 10*C*). There were no intergenotypic differences in the numbers of pHH3+ cells in any region at either age (Fig. 10*D*,*E*). We then measured the proportions of proliferating cells (Ki67+) that were in S-phase (BrdU+), which is an indication of their rate of proliferation (i.e., the longer the cell cycle, the lower the proportion of cells in S-Phase within the defined time window). When we examined all cells, we identified a small but significant decrease at E11.5 in *Pax6^fl/fl^* pTH-R (Fig. 10*F*). When we examined GFP+ cells alone, we found no significant effects of genotype (Fig. 10*G*).

These findings indicate that the loss of both copies of Pax6 in *Zic4*-lineage has a minor effect on proliferation, causing decreases in pTH-C and/or pTH-R at some ages, which was only detectable in the overall population of progenitors and not specifically the *Zic4*-lineage progenitors themselves.

### *Pax6* mutation in *Zic4*-lineage cells increases their contribution to vLGN and dLGN

**Figure 11.**
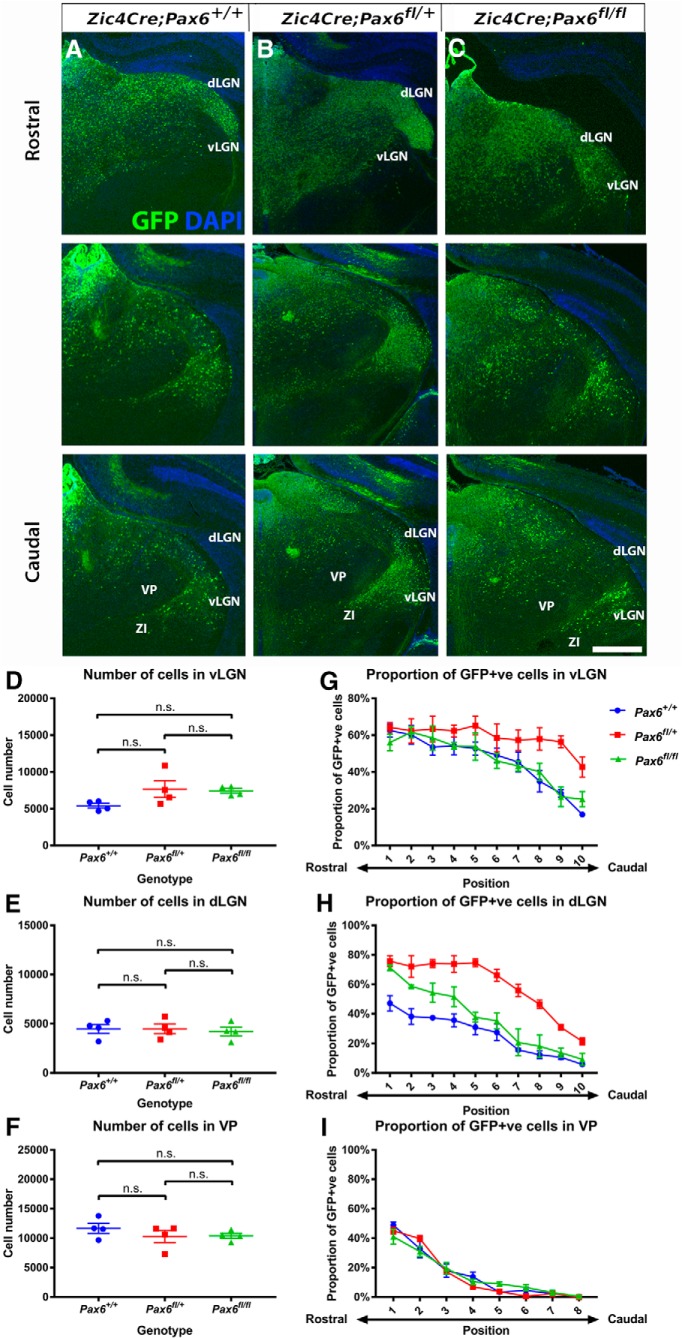
*Pax6* mutation in *Zic4*-lineage cells increases their contribution to vLGN and dLGN. ***A–C***, Sections from rostral to caudal through the diencephalon of P0 *Zic4^Cre^*;*Pax6^+/+^*, *Zic4^Cre^*;*Pax6^fl/+^*, and *Zic4^Cre^*;*Pax6^fl/fl^* embryos. Scale bar: 750 µm. ***D–F***, Total numbers of cells in vLGN, dLGN, and VP in four P0 pups of each genotype; individual values, mean ± SEM are shown. There were no significant effects of genotype. ***G–I***, Proportions of GFP+ (i.e., *Zic4*-lineage) cells in sections equally spaced through the vLGN, dLGN, and VP of P0 *Zic4^Cre^*;*Pax6^+/+^*, *Zic4^Cre^*;*Pax6^fl/+^*, and *Zic4^Cre^*;*Pax6^fl/fl^* pups. In vLGN and dLGN, two-way ANOVA showed significant effects of genotype: vLGN, *F*_(2,90)_ = 23.98, *p* < 0.0001; dLGN, *F*_(2,60)_ = 140.4, *p* < 0.0001. In VP, there were no significant effects. Data are plotted using mean and SEM using at least three pups of each genotype (*n* = 4 for vLGN and *n* = 3 for dLGN and VP).

We then analyzed the effects of *Pax6* mutation in *Zic4*-lineage cells on the numbers of these cells that contribute to vLGN, dLGN, and VP at P0 (Fig. 11). We first estimated the total numbers of cells in each of these nuclei by sampling the densities of all counterstained cells in each nucleus and multiplying by its volume. Volumes were estimated using the ImageJ Volumest plugin on a series of 10 coronal sections with a regular interval of 60 µm from each brain sample. We found no significant intergenotypic differences, indicating that *Pax6* mutation in *Zic4*-lineage cells did not alter overall numbers of cells contributing to these nuclei (Fig. 11*D–F*).

We measured the proportions of cells in each of these nuclei that were *Zic4*-lineage (i.e., GFP+). We found that these proportions were significantly increased in the dLGN of *Pax6^fl/fl^* embryos (Fig. 11*A*,*C*,*H*). They were increased even more in the dLGN of *Pax6^fl/+^* embryos, with more than twice the normal proportions of *Zic4*-lineage cells contributing (Fig. 11*A*,*B*,*H*). Proportions were also increased in the vLGN of *Pax6^fl/+^* embryos (Fig. 11*A*,*B*,*G*). The increases in the dLGN were seen in anterior sections in *Pax6^fl/fl^* embryos and throughout all sections in *Pax6^fl/+^* embryos (Fig. 11*H*). The increases in the vLGN in *Pax6^fl/+^* embryos were greater in more posterior sections (Fig. 11*G*). There were no intergenotypic differences in VP (Fig. 11*I*).

These findings indicate that the dLGN increases its content of *Zic4*-lineage cells at the expense of *Zic4*-non-lineage cells if the *Zic4*-lineage cells are *Pax6^fl/fl^* or *Pax6^fl/+^*. The vLGN increases its content of *Zic4*-lineage cells at the expense of *Zic4*-non-lineage cells if the *Zic4*-lineage cells are *Pax6^fl/+^*.

## Discussion

We have found that *Zic4*-lineage cells normally contribute ∼50% of vLGN cells, ∼25% of dLGN cells, and ∼10% of VP cells at birth. Heterozygosity for a loss-of-function mutation of *Pax6* in *Zic4*-lineage cells greatly increased the contribution that these cells make to the vLGN and dLGN, but not the VP. Homozygosity for the same mutation had a smaller effect on the contribution of *Zic4*-lineage cells to the dLGN and no effect on contribution to vLGN and VP.

These changes are most likely explained by a redistribution of mutant *Zic4*-lineage cells into the dLGN and vLGN. Our findings argue against an alternative explanation involving overproduction of mutant *Zic4*-lineage cells. When we tested whether *Pax6* mutation, either heterozygous or homozygous, had an effect on the early proliferation of specifically the *Zic4*-lineage progenitors, we found none. We did find slight decreases in proliferation in some regions at some ages when we considered the overall populations of progenitors, both *Zic4*-lineage and *Zic4*-non-lineage. These finding suggest that some change in the *Zic4*-lineage cells, perhaps involving altered signaling, had a cell non-autonomous effect on the *Zic4*-non-lineage cells. It does not, however, provide a straightforward explanation for the increased numbers of mutant *Zic4*-lineage cells in the dLGN and vLGN. Another reason that redistribution of postmitotic cells is a more likely mechanism than altered production is that the effects of heterozygosity for *Pax6* mutation on Pax6 protein levels only became obvious in postmitotic cells and not in progenitors.

**Figure 12.**
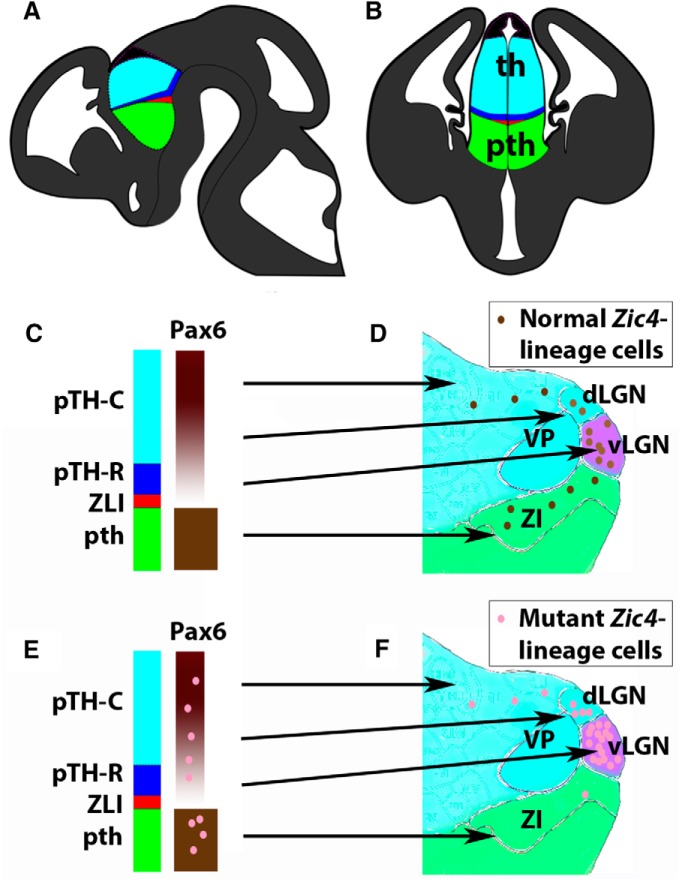
Model. ***A***, ***B***, Sections through the embryonic thalamus (th) and prethalamus (pth) showing the locations of the ZLI (red) and pTH-R (dark blue). ***C***, ***D***, Normally, *Zic4*-lineage cells from progenitors in the ZLI and pTH-R, which express little or no Pax6, contribute to the vLGN. *Zic4*-lineage cells from progenitors in rostral pTH-C (i.e., close to pTH-R), whose levels of Pax6 are relatively low, contribute to dLGN. *Zic4*-lineage cells in prethalamus (pth), which express Pax6 at high levels in both progenitors and postmitotic neurons, contribute to prethalamic regions including the ZI. ***E***, ***F***, Mutant *Zic4*-lineage cells concentrate in the vLGN and/or dLGN, which are derived from thalamic progenitors that normally express little or no Pax6.

Figure 12 summarizes our model. The ZLI and the two progenitor domains posterior to it, pTH-R and pTH-C (Fig. 12*C*,*E*), express substantially different sets of regulatory genes. For example, the ZLI expresses Shh, pTH-R expresses transcription factors such as Nkx2.2 and Asc1, and pTH-C expresses Neurog1 and Neurog2. Several previous fate mapping studies have used genetic tools to exploit these early gene expression patterns to link specific sets of progenitors with mature diencephalic nuclei (Vue et al., 2007, 2009; Delaunay et al., 2009; Jeong et al., 2011; Suzuki-Hirano et al., 2011). These studies showed that the ZLI and adjacent pTH-R contribute to the vLGN, whereas more caudal progenitors in pTH-C contribute progressively to more caudal thalamic nuclei. In other words, the relative positions of thalamic progenitors are well preserved in the spatial arrangement of the nuclei they generate. Pax6 is expressed in a gradient by thalamic progenitors, with its lowest levels in pTH-R and no expression in the ZLI (Fig. 12*C*,*E*). This means that the *Zic4*-lineage cells that distribute to the vLGN and dLGN are derived mainly from progenitors that express little or no Pax6. If homozygous or heterozygous mutation causes *Zic4*-lineage cells to lose or lower their levels of Pax6, then these cells or their daughters preferentially distribute to nuclei that are generated from progenitors that express little or no Pax6. This suggests that the positional information encoded by the levels of Pax6 in diencephalic progenitors is an important determinant of the eventual locations of their daughter cells.

A likely mechanism by which Pax6 levels effect the distribution of diencephalic neurons is through its regulation of cell adhesion molecules that cause cells to sort on the basis of their intercellular interactions. It has long been appreciated in many different systems that cells aggregate if they have similar levels of cadherins (Foty and Steinberg, 2005; Halbleib and Nelson, 2006). In the postnatal mouse brain, different thalamic nuclei express distinct combinations of cadherins: for example, the vLGN and dLGN express Cadherin 5, 8, and 11, whereas VP expresses mainly Cadherin 6 and 11 (Suzuki et al., 1997; Hirano and Takeichi, 2012). Both *in vivo* and *in vitro* studies have shown that Pax6 can regulate molecules such as R-cadherin, L1 cell adhesion molecule and N-CAM (Chalepakis et al., 1994; Edelman and Jones, 1995; Holst et al., 1997; Stoykova et al., 1997; Meech et al., 1999; Andrews and Mastick, 2003; Tyas et al., 2003).

Why *Zic4*-lineage cells heterozygous for a mutation of *Pax6* cause a greater redistribution of *Zic4*-lineage cells affecting both dLGN and vLGN is not clear. One possibility stems from our observation that heterozygosity has a relatively late effect on Pax6 levels in postmitotic neurons, which contribute mainly to the prethalamus. A reduction of Pax6 levels in migrating prethalamic neurons might allow significant numbers of these cells to migrate in an abnormal direction across the boundary from prethalamus into the vLGN and dLGN. The numbers that do this might be greater than the numbers that sort incorrectly following loss of Pax6 from progenitors, as occurs in homozygous deletion. Progenitors might possess a degree of plasticity that allows them to compensate to some degree if they lose Pax6, thereby minimizing the consequences for later sorting of their postmitotic cells.

Whatever the cause of the strong effect of Pax6 heterozygosity, this finding is particularly relevant to human disease caused by *PAX6* haploinsufficiency. In humans, heterozygous loss-of-function mutations affecting *PAX6* cause a disorder with an incidence of ∼1/50,000 live births. Patients show a range of neurologic and psychiatric symptoms including impaired auditory processing, verbal function and social cognition, autism and mental retardation and altered functional connectivity in intrinsic neural networks (Sisodiya et al., 2001; Free et al., 2003; Mitchell et al., 2003; Ellison-Wright et al., 2004; Thompson et al., 2004; Bamiou et al., 2007; Umeda et al., 2010; Pierce et al., 2014). These functional abnormalities are associated with structural defects of the cerebral cortex and its axonal tracts (Sisodiya et al., 2001). Mice with heterozygous loss-of-function mutations of *Pax6* have rarely been used to explore possible pathologies underlying the neurologic and psychiatric symptoms that patients experience. Although one study suggested a slight delay in the onset of corticogenesis in *Pax6*± embryos (Schmahl et al., 1993), other work found no abnormalities of cortical development in these mice (Mi et al., 2013). Almost nothing is reported on the effects of *Pax6*± heterozygosity on development of the diencephalon. Given that the dLGN is the relay nucleus for projections to the visual cortex, the findings of this study suggest that defects of the thalamus and geniculocortical pathway might be found in patients.

## Synthesis

Reviewing Editor: Alfonso Represa, INSERM Decisions are customarily a result of the Reviewing Editor and the peer reviewers coming together and discussing their recommendations until a consensus is reached. When revisions are invited, a fact-based synthesis statement explaining their decision and outlining what is needed to prepare a revision will be listed below. The following reviewer(s) agreed to reveal their identity: NONE. Note: If this manuscript was transferred from JNeurosci and a decision was made to accept the manuscript without peer review, a brief statement to this effect will instead be what is listed below.

Taking into consideration the comments of J Neurosci reviewers and taking into consideration the quality of the work and the interest of the study, I consider the paper suitable for publication in eNeuro
